# The acute toxicity of chemically and physically dispersed crude oil to key arctic species under arctic conditions during the open water season

**DOI:** 10.1002/etc.2307

**Published:** 2013-08-14

**Authors:** William W Gardiner, Jack Q Word, Jack D Word, Robert A Perkins, Kelly M McFarlin, Brian W Hester, Lucinda S Word, Collin M Ray

**Affiliations:** † NewFields NorthwestPort Gamble, Washington, USA; ‡ Institute of Northern Engineering, University of Alaska FairbanksFairbanks, Alaska, USA

**Keywords:** Arctic, Alaska North Slope crude oil, Chemically dispersed oil, *Calanus glacialis*, *Boreogadus saida*

## Abstract

The acute toxicity of physically and chemically dispersed crude oil and the dispersant Corexit 9500 were evaluated for key Arctic species. The copepod *Calanus glacialis*, juvenile Arctic cod (*Boreogadus saida*), and larval sculpin (*Myoxocephalus* sp.) were tested under conditions representative of the Beaufort and Chukchi Seas during the ice-free season. The toxicity of 3 water-accommodated fractions (WAF) of Alaska North Slope crude oil was examined with spiked, declining exposures. A dispersant-only test was conducted with the copepod *C. glacialis*. Each preparation with oil (WAF, breaking wave WAF [BWWAF], and chemically enhanced WAF [CEWAF]) produced distinct suites of hydrocarbon constituents; the total concentrations of oil were lowest in WAF and highest in CEWAF preparations. The relative sensitivity for the different species and age classes was similar within each WAF type. Median lethal concentration values based on total petroleum hydrocarbons ranged from 1.6 mg/L to 4.0 mg/L for WAF and BWWAF treatments and from 22 mg/L to 62 mg/L for CEWAF. For Corexit 9500 exposures, median lethal concentration values ranged from 17 mg/L to 50 mg/L. The differences in the relative toxicity among the accommodated fractions indicated that the majority of petroleum hydrocarbons in the CEWAF are in less acutely toxic forms than the components that dominate the WAF or BWWAF. Further evaluation showed that the parent polycyclic aromatic hydrocarbon compounds, specifically naphthalene, were highly correlated to acute toxicity. *Environ Toxicol Chem* 2013;32:2284–2300.

## INTRODUCTION

An extended ice-free season has expanded opportunities for oil and gas exploration in the Beaufort and Chukchi Seas and has increased the need to better understand the relative consequences of oil spill response alternatives under arctic conditions. Chemical dispersants are oil-spill countermeasures that reduce the surface tension of oil and cause formation of small droplets that facilitate rapid movement of oil from the surface into the water column. In 2008, a workshop was held in Anchorage, Alaska to identify critical gaps in knowledge regarding the use of dispersants in the Beaufort and Chukchi Seas 1. A panel of experts, including representatives from government, academia, the North Slope Borough, and industry, concluded that data on the relative toxicity of crude oil and dispersed oil to pelagic and epipelagic organisms in the Arctic were limited and that further toxicity data were needed to evaluate the net environmental consequences of dispersants used in open waters. Although the toxicity of physically and chemically dispersed oil has been documented for nonarctic species including temperate, tropical or sub-arctic organisms 2–5, less is known about the sensitivity of species indigenous to Arctic Seas. The goal of the present study was to characterize the acute toxicity of Alaska North Slope (ANS) crude oil and dispersed oil to 3 key food web species living in ice-free waters of the Beaufort and Chukchi Seas. This data can be used to support evaluations of the net environmental consequences associated with dispersant use on multiple environmental compartments in the Arctic.

During ice-free periods, the open waters of the Beaufort and Chukchi Seas are typically characterized by temperatures ranging from 0 °C to 5 °C and salinities varying from 28‰ to 35‰. The low water temperatures and varying salinities affect the behavior and toxicity of hydrocarbons, altering petroleum viscosity and hydrocarbon solubility and volatilization 6,7. Furthermore, the fate of physically and chemically dispersed petroleum in open waters is affected by diffusion and dilution into the underlying water column. Field samples collected from beneath oil slicks indicate that petroleum transferred into the water column by diffusion, surface disturbance, or dispersant application is mixed with underlying water by turbulent or eularian mixing. This results in lower petroleum concentrations in the water column than are present at the surface 8. After the initial dilution into the water column occurs, lagrangian (lateral) movement results in longer-term exposures of water column organisms to lower petroleum concentrations. The initial eularian mixing results in a rapid decrease in hydrocarbon concentrations in the water column, falling to below detection limits within a matter of hours following dispersant application 9–14. These field studies indicate that the exposure of pelagic species in open waters decreases over time and that toxicity tests using spiked or declining exposures emulate field conditions more effectively than continuous laboratory exposures.

Cold-water pelagic species that are potentially exposed to dispersed oil have behavioral, morphological, and physiological adaptations that allow them to tolerate the environmental extremes of the Arctic Ocean. Polar fish and invertebrates have lower metabolic rates and are generally thought to live longer than their non-arctic counterparts 7. Cold-water species also have metabolic adaptations that allow for proper muscle and cardiac function 15,16 and antifreeze proteins that prevent ice-crystal formation in blood 17,18. Arctic copepods have exceptionally high oil content that varies seasonally, storing wax esters that are consumed during overwintering periods when food resources are limited 19,20. These adaptations have been cited as indications that the responses of Arctic species may differ from those of temperate species used in previous tests with physically and chemically dispersed petroleum 7.

The pelagic communities in the Beaufort and Chukchi seas have relatively simple food webs that change dramatically during the short ice-free season. Copepods, euphausiids, and krill represent the heterotrophic base of the Arctic food web, supporting not only Arctic fish species, such as Arctic cod, but also important migratory species such as baleen whales and sea birds 21–24. Calanoid copepods are numerically dominant zooplankton and several species, including *Calanus glacialis* and *Calanus hyperborialis* are present in the Arctic system throughout the year. Arctic cod, *Boreogadus saida*, are the most widespread and abundant fish species in the Chukchi and Beaufort Seas 25. They represent a critical link between zooplankton and higher order predators 26 as the most dominant prey species for marine sea birds, seals, and beluga whales 27–29. Both copepods and Arctic cod were species that the workshop participants identified as valuable ecosystem components in the Beaufort and Chukchi Seas, and both were considered suitable for laboratory evaluations.

The present study examines the median lethal concentrations for 3 wild-caught arctic species exposed to physically and chemically-dispersed crude oil. Results are reported for measured total petroleum hydrocarbons (TPH), measured polycyclic aromatic hydrocarbons (PAHs), and percentage of dilution of the accommodated fractions. These data were then used to evaluate the relative sensitivity of the three arctic species and to compare the observed toxicity differences between water-accommodated fraction (WAF) treatments. To gain insight into the potential impacts of dispersed oil, toxicity data from the present study were compared with concentrations that have been measured in the water column following dispersant application during spill events.

## METHODS

Acute toxicity experiments followed laboratory protocols adapted from the Chemical Response to Oil Spills Ecologic Research Forum (CROSERF) 4 with modifications to better represent ice-free conditions of the Beaufort and Chukchi Seas 2,3,30–35. Modifications incorporated in this testing program included: 1) test species that are representative of arctic food webs at age classes representative of ice-free period; 2) Beaufort/Chukchi Sea seawater at temperatures representative of ice free-period (−1 °C to 5 °C); 3) oil-to-water and dispersant-to-oil loading rates representative of potential field conditions; 4) use of spiked exposures simulating changes in exposure concentrations that occur in a real-world oil discharge; 5) use of extended test duration to ensure an accurate assessment of acute toxicity responses by arctic species to spiked exposures; and 6) comprehensive analytical characterization of WAFs to quantify exposure concentrations including TPHs, and total polynuclear aromatic hydrocarbons (parent and alkylated compounds) for every test. These method refinements are discussed briefly in the following paragraphs.

### Test organisms

Three species playing key roles in arctic food webs were selected for acute toxicity testing: Calanoid copepods (*C. glacialis*), juvenile Arctic cod (*B. saida*), and larval sculpin (*Myoxocephalus* sp.). All test organisms were collected from the Beaufort or Chukchi Seas and represented age classes present in the upper 10 m of the water column during the ice-free season. *Calanus* sp. copepods dominate the meso-zooplankton in the open water seasons and are one of the primary food sources for many higher trophic level species. *Calanus glacialis* were collected using plankton ring-nets in both the early open-water season (July and August) and late open-water season (October and November) and tested in the late copepodite or mature stages (3.6–5.5 mm).

Arctic cod are the most abundant fish species, occupying Arctic waters year-round. Although eggs and early larval forms (yolk sac stage) of Arctic cod are present under ice between December and February off Barrow, Alaska 36,37, there is a low abundance of early larval stages during the open-water season 38. However, during the early summer (July and August), juvenile Arctic cod are present in near shore areas. Juvenile Arctic cod (60–30 mm) were collected at the entrance to Elson Lagoon using a Fyke net during the early open-water season (August). Arctic cod were tested as juveniles, with sizes ranging from 60 mm to 130 mm.

On the other hand, larval sculpin were available during the open-water, ice-free period and were present in early larval (yolk sac) development stages during the summer season. Sculpin are among the most speciose fish taxa in the Arctic 39, are an important food resource for fish, marine mammals, and seabirds 40 and represent a sensitive early life stage for this testing program. Larval sculpin (8–15 mm) were collected from Elson Lagoon in July and August using a beach seine net. Tests with the larval sculpin were conducted approximately 2 wk apart; one test used yolk-sac larvae and the second test used post-yolk-sac larvae.

All test organisms were transferred directly to the Barrow Arctic Sciences Center facility where they were acclimated and held in 10 gallon or 20 gallon aquaria in natural seawater at 2 °C. Copepods, cod, and larval sculpin were fed and were observed feeding on natural plankton supplemented with algal paste or *Artemia* sp. nauplii during acclimation and were held a minimum of 1 wk prior to testing. Strict adherence to test specific guidelines approved by the Institutional Animal Care and Use Committee at the University of Alaska Fairbanks insured the humane treatment of all test organisms.

### Preparation of stock solutions and dilution series

Three types of test solutions were used to assess test organism responses to petroleum and chemically dispersed petroleum treatments. The WAF represented physical dispersion of oil into the water column with moderate physical disturbance, emulating nonbreaking wave field conditions that would not produce emulsions, yielding primarily soluble petroleum compounds 41. Chemically enhanced accommodated fractions represented chemical and physical dispersions of oil, with a similar mixing energy as the WAF, producing small dispersed oil droplets and water soluble fractions of oil. A third method (termed breaking-wave WAF [BWWAF]) was a physical dispersion (also in the absence of a dispersant) representing a slightly more energetic sea state in which small to large nonbreaking wavelets predominate in the presence of light to gentle breezes (Beaufort sea state of 3–4). This procedure was expected to produce higher quantities of oil within the water column than the WAF physical dispersions but less than the chemically enhanced preparations.

Test solutions were prepared using fresh ANS crude and filtered Beaufort or Chukchi seawater. Alaska North Slope is medium-grade oil with an American Petroleum Institute gravity of approximately 29.6; this oil was collected from the Valdez Marine Terminal in July 2009, supplied by Alyeska Pipeline Service Company. The hydrocarbon profile of ANS crude oil ranges from 53% to 87% aliphatic hydrocarbons (i.e., straight-chained or branched) and from 10% to 37% aromatic hydrocarbons (i.e., fraction having benzene ring(s). Dispersed test solutions were prepared using Corexit 9500 42. All test solutions were prepared in 20-L glass aspirator bottles, fitted at the bottom with a glass port and Teflon ball valve that allowed the preparations to be collected from the bottom to avoid entrainment of surface oil. Test solutions were prepared with 16 L of aerated, 0.45-μm filtered seawater with 20% headspace. The seawater was allowed to acclimate to test conditions in a 2 °C cold-room. A Teflon-coated magnetic stir bar was placed in the aspirator bottle set on a magnetic stir plate and activated to produce a 20% vortex. Petroleum was added gravimetrically at a loading rate of 10 g/L (1:100). For each batch of test water, 160 g of ANS was poured into a tared beaker and then added directly into the aspirator bottle near the margin of the vortex, avoiding any contact with the walls of the aspirator bottle. For the dispersant preparations, Corexit 9500 was added after the oil at a dispersant-to-oil ratio of 1:20. For 160 g of oil, 8 g of dispersant was added directly into the aspirator bottle. For the dispersant-only preparation, 8 g of dispersant was added to 16 L of seawater. Aspirator bottles were sealed, and mixing speeds were adjusted to obtain a 20% to 25% vortex for all test solutions (14 *g*; ∼300 rpm). Test solutions were mixed in the dark at test temperature (2 °C) for 18 h, followed by a 6-h phase-separation or resting period with no mixing.

The BWWAF was prepared in a manner similar to the WAF, with the exception that periods of increased turbulence were introduced by manual agitation for 1 min at 15-min intervals during the first 2 h of mixing and during the last hour of mixing prior to the resting period. Increased mixing was achieved by manually rocking the aspirator bottle, creating particle collisions with the walls and bottom of the carboy, which resulted in a chaotic mixing energy and served to break apart larger globules of oil. Mixing with electromagnetically controlled stir bars maintained the 20% to 25% vortex during intervals between shaking (14 *g*; ∼300 rpm).

Stock solutions for test treatments and samples for initial chemical analyses were collected at the end of the resting phase. This allowed larger droplets not entrained in the water column to float to the surface, removing them from the test solutions used for toxicity tests and analytical chemistry. Aliquots for chemical analyses were transferred directly into pre-cleaned 1-L amber glass bottles and held at 2 °C prior to analysis. Stock solutions were distributed into individual 8-L aspirator bottles for preparation of the WAF, chemically enhanced WAF (CEWAF), and BWWAF dilution series. Test dilutions were prepared using variable dilution, making each test concentration individually by adding the appropriate amount of seawater to the 100% stock solution. In each test, 5 or 6 dilutions were typically included, with a concurrent seawater-only control. In some tests, however, reduction to 4 dilutions allowed WAF and BWWAF treatments to be evaluated during the same experiment. Depending on WAF treatment response, the dilution series investigated were 100%, 50%, 25%, 12.5%, 6.25%, and 3.125% or 100%, 40%, 16%, 4%, and 1%. Subsamples for analysis were shipped on dry-ice via overnight delivery to the analytical chemistry laboratory, Alpha Analytical in Westborough, Massachusetts, USA.

### Test design

Singer et al. 2, Fuller et al. 5, and the National Research Council 43 found that constant exposure testing is likely to overestimate toxicity that occurs in the open ocean beneath a spill with or without application of dispersants. Spiked exposures with declining concentrations targeting a specific rate of reduced concentrations were found to more closely represent the natural dilution of dispersed crude oil in the water column 5,44. Singer et al. 2 used a flow rate sufficient to reach nondetect levels (0.3 ppm) over 8 h to evaluate effects to 4 temperate invertebrates after a spiked exposure to the dispersant Corexit 9527 and oil. However, it has been reported that for large spills or in areas with limited circulation, higher concentrations of dispersed oil may persist for a longer period of time 43. To simulate the dilution observed under oil spills in the field, spiked exposures were conducted with concentrations theoretically declining by half every 4 h in the present study.

Copepod and larval sculpin tests were conducted with 300 mL of test solution in 500-mL glass jars fitted with a screened exit port. Seawater was supplied using peristaltic pumps or by direct replacement using a Zumwalt device 45. Arctic cod were tested with 5 L of test solution in 2-gallon glass aquaria with static renewal of 2.5 L every 4 h. All tests were conducted with 4 replicate chambers per treatment and with 5 test organisms per chamber. Toxicity tests were conducted at 2 °C in open test systems to allow for volatilization, as would occur in open water.

Six copepod experiments were conducted during the early open-water season, and 5 experiments were conducted with the late-season copepods. Breaking wave WAF treatments were not included in tests conducted with late, ice-free season copepods; the BWWAF procedure was added after this testing round had occurred. Two Corexit 9500-only tests were conducted with the early-season copepods. Four tests were conducted with juvenile Arctic cod. Four tests were also conducted with larval sculpin; the first 2 tests with the sculpin were with larvae that, based on size and development stage, were approximately 14 d old (yolk-sac stage). The second 2 tests were conducted with 28 d old (post-yolk-sac) larvae.

Previous research has indicated that arctic species may require an extended test period beyond the standard test duration of 96 h to exhibit responses to a spiked petroleum exposure 7,46,47. Based on an evaluation of control mortality and dose responses in preliminary tests 48, copepod tests were conducted as 12-d tests (a standard 96-h spiked exposure followed by an additional 8-d observation period) to detect responses exhibited over a longer period of time. However, during initial tests, no significant additional effects were observed for either fish species over an extended test period. Therefore, the test duration was 96 h for the fish; all median lethal concentration (LC50) evaluations for fish were based on this standard test duration. The test endpoint was mortality, as defined by a lack of movement with gentle probing. Copepods were fed a mixture of algal paste and live algae prior to test initiation, at 48 h, and daily during the observation period. Fish species were fed field-collected plankton or *Artemia* sp. nauplii during the test.

Dissolved oxygen, temperature, salinity, and pH were measured at test initiation in all test chambers and monitored daily in a surrogate chamber per treatment during the experiments. All tests were conducted at 2 °C ± 1 °C. Dissolved oxygen levels remained above 60% saturation throughout the tests and pH ranged from 7.2 to 8.3. Salinity was within ±2‰ of ambient (30–35‰).

### Chemical analysis

Chemical analysis is critical to understanding toxicity of the CEWAF and WAF. While many laboratory studies report results based on nominal oil loadings or percentage of WAF, such results are difficult to link to measured exposure concentrations in the field during spill events, which are typically expressed as measured hydrocarbon concentrations (mg/L or μg/L). Therefore, petroleum hydrocarbon concentrations were measured in whole-water (unfiltered) test solutions to allow dose–response relationships to be established that enable comparisons with actual field exposure concentrations.

Chemical analyses were conducted by Alpha Analytical. Analytical chemistry was performed for all stock solutions prepared during the present study, as well as all dilutions prepared during the first 5 test batches. All samples were analyzed for 27 parent PAHs and 43 alkylated PAHs (Supplemental Data, Table 1) by gas chromatography–mass spectrometry (GC-MS) using a modified US Environmental Protection Agency (USEPA) method 8270C 49,50. The target 70 PAH analytes were quantified using average response factors generated from the 5-point instrument calibration relative to the internal standards. Alkylated PAH concentrations were determined using the average response factors for the corresponding parent compound 51. Total PAHs are reported as the sum of parent and alkylated PAHs (the bicyclic decalin parent and alkylated compounds are included in this summation). All samples were analyzed for TPH (C_9_–C_44_) by gas chromatography/flame ionization detector using a modification of USEPA Method 8015 52,53. As with the GC-MS, quantification was accomplished using the average response factors generated from the 5-point instrument calibration relative to the internal standards. An aliquot of fresh ANS crude oil was also analyzed concurrent with the first group of samples. Quality assurance and quality control procedures included an analytical blank, laboratory control sample and duplicate, matrix spike and duplicate, and standard reference material. Corexit 9500 exposure concentrations were calculated based on loading rates and dilutions of the 100% nominal concentration. Based on the uniform appearance of the dispersant-only preparation, the lack of a slick or concentrated layer at the surface of the mixing container, and the hydrophilic properties of Corexit 9500, the test solution was considered to be well mixed.

### Statistical analyses

Lethality was assessed in terms of percentage of mortality for each replicate; means and standard deviations were determined for each test treatment representing a 96-h (fish) or 12-d (copepod) test duration, with an initially spiked and a declining dose of test media. These data were evaluated based on measured concentrations of petroleum hydrocarbons at test initiation; TPH represented the sum of aromatic and aliphatic hydrocarbons (C_9_–C_44_), and TPAHs were the sum of parent PAHs and their alkylated homologs. Statistical analyses were performed using Comprehensive Environmental Toxicity Information System software 54. Prior to statistical comparison, data were tested for normal distribution. When data violated the assumption of a normal distribution, they were transformed using an arcsine square root transformation prior to statistical analysis. Median lethal concentration values representing either 12-d or 96-h spiked exposures (copepod and fish tests, respectively) were calculated for each test treatment for each round of testing, as well as for a dataset that included the results from all experiments. Median lethal concentration values were generated by probit analysis if data were normally distributed or the trimmed Spearman–Karber technique 55 for non-parametric distributions. Data were control normalized for point estimates using an Abbott's formula 56. A response threshold, or threshold dose, was determined for each dose–response relationship using the combined dataset of all experiments for each species. The response threshold was defined as the concentration that resulted in 10% lethal concentration (LC10) based on a polynomial distribution of the combined dataset 57.

## RESULTS

**Figure 1:**
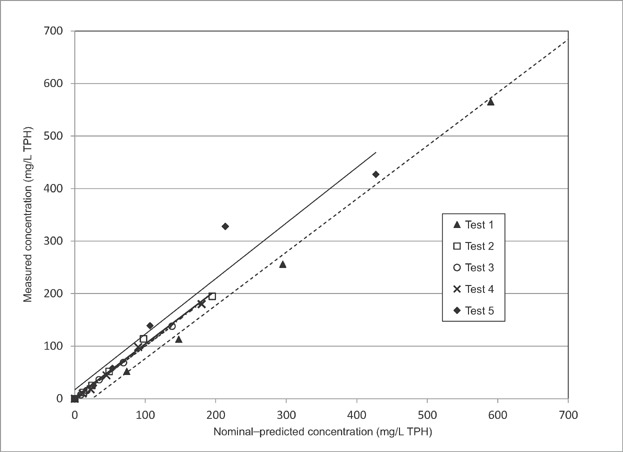
Measured total petroleum hydrocarbons (TPH) concentrations compared to nominal concentrations in initial chemically enhanced water-accommodated fractions preparations for 5 copepod tests conducted in 2009. Regression lines shown for each test series; *p* < 1.6 E-3 and *r*^2^ > 0.94 for all tests.

Analytical chemistry results demonstrated that nominal concentrations were not predictive of the measured concentrations produced in 100% WAFs. Both TPH and TPAH concentrations were variable between batches with similar loading rates. However, once the 100% solutions of WAF or CEWAF were produced, hydrocarbon concentrations in subsequent dilutions proved to be highly predictable. Chemical analysis was conducted for all dilutions of the first 5 batches of WAF and CEWAF. The measured TPH concentration in each of the dilutions was highly correlated to the predicted concentration based on the nominal dilution of the initial 100% stock solution (Figure 1). This was also true for the various PAH constituents. Based on the demonstrated agreement between the nominal and measured concentrations for the dilutions in the first 5 batches, the concentration for the dilutions of WAF, BWWAF, and CEWAF in subsequent tests were calculated based on the product of the measured concentration in the WAF stock solution and the percentage of WAF dilution.

### TPH and TPAH profiles in stock solutions of WAF, BWWAF, and CEWAF

Total petroleum hydrocarbons in the initial 100% WAF preparations ranged from 0.80 mg/L to 4.03 mg/L, with a mean TPH concentration of 1.95 mg/L (±1.15 standard deviation [SD]; Table 1). The mean TPAH concentration in the WAF preparations was 0.067 mg/L (±0.022 SD), while the mean concentration of naphthalene was 0.030 mg/L (±0.010 SD) in WAF stock solution (∼45% of the total PAH compounds).

**Table 1:** Petroleum hydrocarbon concentrations in undiluted water-accommodated fractions (WAFs) measured at test initiation

Class	Mean (±SD), mg/L			Relative percentage contribution to TPAH		
	WAF (*n* = 11)	BWWAF (*n* = 6)	CEWAF (*n* = 12)	WAF	BWWAF	CEWAF
TPH	1.95 (±1.15)	6.51 (±1.94)	379 (±253)			
TPAH	0.067 (±0.022)	0.191 (±0.047)	8.00 (±5.38)			
∑ Parent PAHs	0.034 (±0.011)	0.069 (±0.014)	1.03 (±0.370)	50%	38%	13%
∑ Alkylated PAHs	0.033 (±0.011)	0.122 (±0.033)	6.97 (±0.5.01)	50%	62%	87%
Parent naphthalene	0.030 (±0.010)	0.058 (0.012)	0.334 (±0.127)	45%	31%	4%

SD = standard deviation; TPAH = total polynuclear aromatic hydrocarbons; BWWAF = breaking wave water-accommodated fraction; CEWAF = chemically enhanced water-accommodated fraction.

With the increased mixing energy in BWWAF preparations simulating a Beaufort sea state of 3 to 4, total oil concentrations increased, and TPH concentrations were higher than those measured in WAF preparations, ranging from 4.1 mg/L to 13.0 mg/L. The mean TPAH concentration in the BWWAF was 0.191 mg/L (±0.047 SD), more than twice the concentration of TPAH in WAF treatments. The relative distribution of PAHs in the BWWAF preparation was similar to the WAF in that the naphthalene and alkyl-naphthalenes comprised the majority of TPAH. The mean concentration of parent naphthalene was 0.058 mg/L (±0.012 SD), representing approximately 31% of the total PAHs. However, the alkylated homologs represented a slightly higher fraction of the TPAH than parent compounds in the BWWAF preparation.

**Figure 2:**
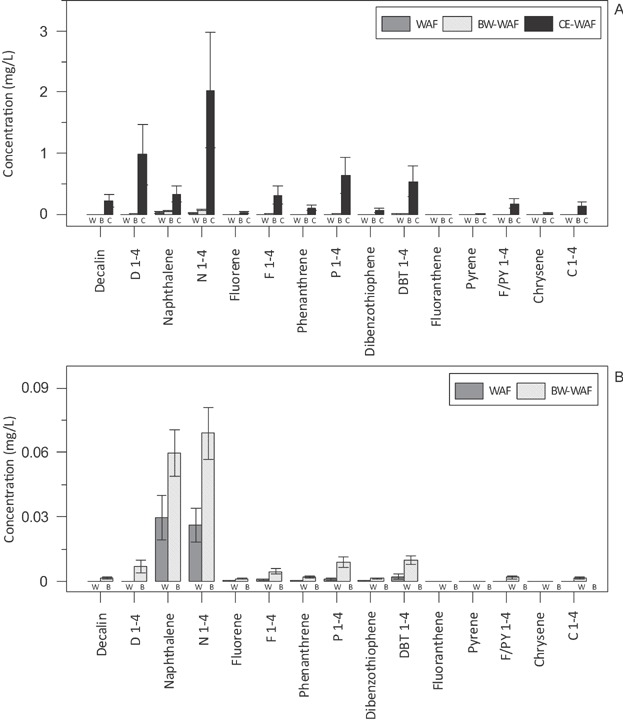
Concentrations of parent and alkylated polycyclic aromatic hydrocarbons in undiluted water-accommodated fractions (WAFs) for all preparations (A) and physically dispersed petroleum preparations (B). (Error bars represent the standard deviation α = 0.05.) BWWAF = breaking wave WAF; CEWAF = chemically enhanced WAF.

In comparison, concentrations of TPH in CEWAF stock solutions were substantially higher and more variable than in the two physically dispersed preparations (Figure 2). Total petroleum hydrocarbon concentrations ranged from 138 mg/L to 1180 mg/L with a mean of 379 mg/L (±253 SD). Total PAH in the CEWAF was also substantially higher than physically dispersed treatments, with a mean of 8.00 mg/L (±5.38 SD; Table 1). Despite TPAH concentrations that were up to 100-fold higher in the CEWAF than the WAF, the relative concentration of parent PAHs in the CEWAF was less than the WAF and BWWAF. The average concentration of parent PAHs and alkylated PAHs in the CEWAF preparations was 1.03 mg/L and 6.97 mg/L, respectively, demonstrating that CEWAF preparations were dominated by alkylated PAHs (87% of the total) and were more representative of the composition of the ANS crude oil.

**Figure 3:**
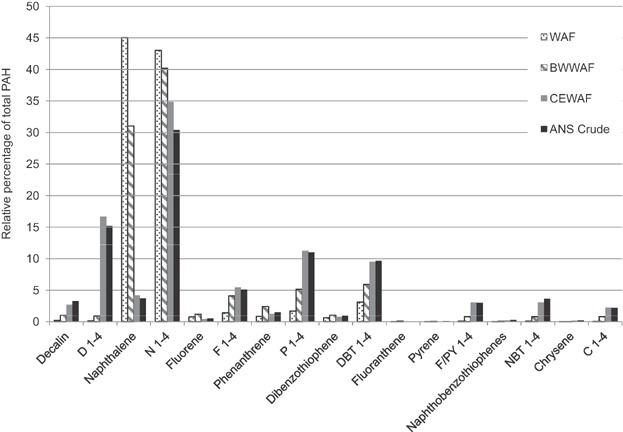
Mean percentage of parent and alkylated polycyclic aromatic hydrocarbons (PAHs) in test water-accommodated fractions (WAF) and fresh Alaska North Slope (ANS) crude oil. BWWAF = breaking wave WAF; CEWAF = chemically enhanced WAF.

Although naphthalene was the dominant parent PAH compound in CEWAF preparations with a mean concentration of 0.334 mg/L (±0.127 SD), it represented only 4.2% of the total PAHs present (Figure 3). Alkylated naphthalenes (C1–C4) represented 35% of the TPAH, with alkylated fluorene, phenanthrene, dibenzothiophene, and the bicyclic decalin compounds, representing an additional 52% of the total PAH. Few high molecular weight PAHs were observed in the CEWAF aside from small peaks of C1 through C4 fluoranthene, pyrene, naphthobenzothiophene, and chrysene (Figure 2). The relative distribution of PAHs in the CEWAF was similar to the hydrocarbon profile of the fresh ANS crude oil used in these studies. Polycyclic aromatic hydrocarbons in the fresh ANS crude oil were also dominated by the alkyl-naphthalenes, comprising 30% total, whereas the predominant parent compound, naphthalene, comprised 4% of the total. The alkylated homologs of fluorene, phenanthrene, and dibenzothiophene and the bicyclic decalin compounds comprised an additional 57% of the TPAH in the fresh ANS crude, with parent PAHs comprising the remaining 7% of the total. As with the CEWAF, few high molecular weight PAHs were observed in the fresh ANS crude oil.

The difference in the relative contributions of analytes in the various accommodated fraction preparations reflects the nature of the compounds introduced into the water. The low-energy WAF preparation was dominated by the more soluble components of the ANS oil with the less soluble components remaining on the water surface. The additional energy associated with the BWWAF further increased the concentration of the more soluble components and added small amounts of less soluble components, but the majority of oil still remained on the surface of the water. The CEWAF chemical profile was the same as the released oil, indicating that the dispersant effectively transferred oil from the surface into the water column. This was also indicated by the presence of dispersed oil droplets in the water column following the settling period.

### Acute toxicity in Arctic marine species

Test performance of all laboratory experiments met acceptability criteria based on control survival at test termination (≥80% survival). Mean percentage of survival in controls from copepod experiments ranged from 85% to 98% at 12 d with one exception (a copepod test with a mean control survival of 78% was included in our analysis because the increased biological responses were similar to other treatments). Mean control survival at 96 h was above 90% for the 4 tests conducted with juvenile Arctic cod. Mean control survival of the 4 experiments with larval sculpin ranged from 80% to 100% at 96 h. The results for all tests are summarized in Table 2 and Table 3.

**Table 2:** Summary acute toxicity test results for three arctic species based on total petroleum hydrocarbon analyses conducted at test initiation (mg/L)

Test	CEWAF		BWWAF		WAF	
	Mean lethal dilution (%)	LC50	Mean lethal dilution (%)	LC50	Mean lethal dilution (%)	LC50
12-d tests with *Calanus glacialis* (Early open-water season)						
1	6.2	15	55.8	2.4	NC	NC
2	10.4	18	68.2	3.3	NC	NC
3	9.4	37	79.0	4.0	NC	NC
4	6.8	30	100	NC	NC	NC
5	8.4	16	61.2	5.0	NC	NC
6	7.1	14	44.6	4.0	NC	NC
Mean	8.0	22	68.1	4.0	NC	NC
SD	1.5	9.5	17.7	1.1	NC	NC
12-d tests with *Calanus glacialis* (Late open-water season)						
1	14.3	75	NT	NT	NC	NC
2	28.8	50	NT	NT	NC	NC
3	44.0	75	NT	NT	NC	NC
4	22.4	30	NT	NT	NC	NC
5	20	79	NT	NT	NC	NC
Mean	25.9	62	NT	NC	NC	NC
SD	10.2	21	NT			
96-h tests with *Boreogadus saida*						
1	10.0	80	48.8	1.6	51.6	1.5
2	14.6	46	10.0	5.7	44.7	2.0
3	15.6	45	53.9	2.5	59	1.2
4	11.9	50	NT	NT	NT	NT
Mean	13	55	37.6	3.3	51.8	1.6
SD	2.6	17	24.0	2.2	7.2	0.4
96-h tests with *Myoxocephalus* sp						
1	4.2	18	51.0	2.2	100	1.4
2	3.8	17	56.5	3.0	78.4	1.6
3	8.0	29	59.5	5.1	85	3.0
4	7.0	46	59.5	5.7	83.5	3.3
Mean	5.8	28	56.6	4.0	85.1	2.3
SD	2.0	14	4.0	1.7	10.3	1.0

NC = not calculable; NT = not tested; LC50 = median lethal concentration; TPH = total petroleum hydrocarbons; WAF = water-accommodated fraction; BWWAF = breaking wave water-accommodated fraction; CEWAF = chemically enhanced water-accommodated fraction; SD = standard deviation.

**Table 3:** Summary of acute toxicity results for three arctic species based on total PAH concentrations (parent or alkylated forms) measured at test initiation

Test	CEWAF		BWWAF		WAF	
	LC50 (mg/L PAH)		LC50 (mg/L PAH)		LC50 (mg/L PAH)	
	∑ Parent	∑ Alkyl	∑ Parent	∑ Alkyl	∑ Parent	∑ Alkyl
12-d test with *Calanus glacialis* (Early open-water season)						
1	0.04	0.24	0.03	0.04	NC	NC
2	0.07	0.43	0.03	0.06	NC	NC
3	0.11	0.62	0.06	0.11	NC	NC
4	0.09	0.51	NC	NC	NC	NC
5	0.04	0.26	0.03	0.04	NC	NC
6	0.03	0.18	0.03	0.04	NC	NC
Mean	0.06	0.37	0.03	0.06	NC	NC
SD	0.03	0.17	0.01	0.02		
12-d test with *Calanus glacialis* (Late open-water season)						
1	0.03	1.67	NT	NT	NC	NC
2	0.13	0.80	NT	NT	NC	NC
3	0.17	0.63	NT	NT	NC	NC
4	0.06	0.31	NT	NT	NC	NC
5	0.23	1.58	NT	NT	NC	NC
Mean	0.13	1.00	NT	NT	NC	NC
SD	0.08	0.60				
96-h test with *Boreogadus saida*						
1	0.31	1.97	0.06	0.07	0.04	0.03
2	0.13	0.94	0.05	0.1	0.04	0.04
3	0.15	0.96	0.04	0.06	0.02	0.03
4	0.38	2.67	NT	NT	NT	NT
Mean	0.24	1.64	0.05	0.08	0.03	0.03
SD	0.12	0.84	0.01	0.02	0.01	0.00
96-h test with *Myoxocephalus* sp						
1	0.10	0.38	0.02	0.05	0.02	0.02
2	0.07	0.25	0.03	0.06	0.03	0.03
3	0.17	0.68	0.06	0.09	0.04	0.04
4	0.32	1.28	0.07	0.11	0.04	0.04
Mean	0.16	0.93	0.05	0.08	0.04	0.03
SD	0.11	0.66	0.02	0.03	0.01	0.01

WAF = water-accommodated fraction; BWWAF = breaking wave water-accommodated fraction; CEWAF = chemically enhanced water-accommodated fraction; LC50 = median lethal concentration; SD = standard deviation; NC = not calculable, undiluted WAF did not show significant toxicity; NT = not tested.

### Copepods: 12-d tests representing early ice-free season

**Figure 4:**
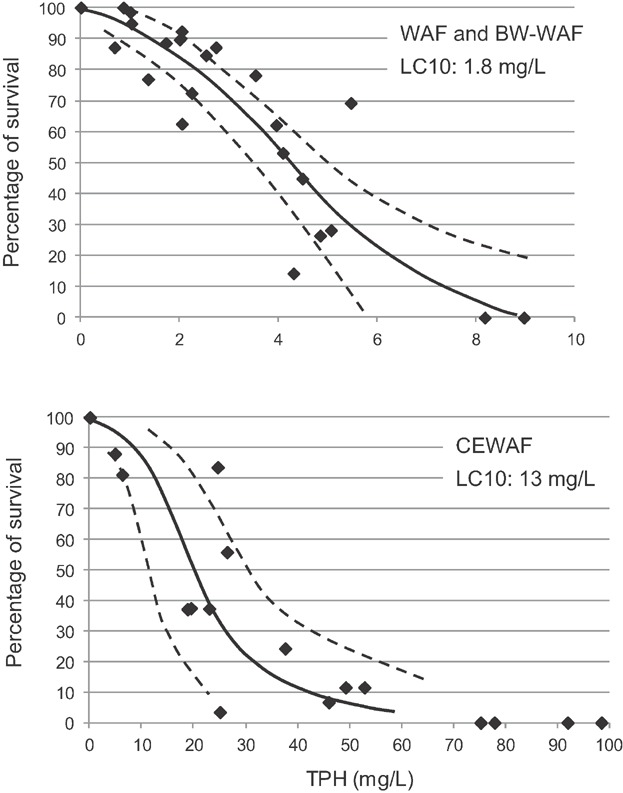
Concentration response curves for early season copepod tests with physically dispersed (water-accommodated fractions [WAF] and breaking wave WAF [BWWAF]; *r*^2^ = 0.88) and chemically dispersed Alaska North Slope (ANS) crude oil (chemically enhanced WAF [CEWAF]; *r*^2^ = 0.87) (control normalized data; dashed lines represent 95% confidence intervals).

There were no calculable LC50 values for the WAF exposures and no statistically significant differences in survival between the 100% WAF and control treatments. However, as the greater mixing energies created higher exposure concentrations, there was a significant increase in lethality for the BWWAF exposures. Based on WAF dilutions, the mean lethal dilution for the BWWAF was 68.1% (±17.7 SD). The mean 12-d LC50 for measured TPH was 4.0 mg/L TPH (±1.1 SD). Data compiled on all data from the 6 copepod tests with physical dispersions (WAF and BWWAF) produced a steep dose–response curve with an LC10 response threshold of 1.8 mg/L (Figure 4).

Mortality was observed in all CEWAF experiments, with statistically significant decreases in survival in the 100% CEWAF treatments. The dispersants were effective in moving oil droplets into the water column, increasing exposure concentrations in the 100% CEWAF. The mean lethal dilution for CEWAF exposures was 8.0% (±1.5 SD), and the mean LC50 was 22 mg/L (±9.5 SD) based on TPH. Summarizing all data from the 6 tests, the LC10 response threshold for the CEWAF dose–response curve was 13 mg/L TPH (Figure 4). The LC50 for TPH in CEWAF was nearly 5-fold higher than the mean LC50 for the physical dispersions (WAF and BWWAF).

The LC50 values for TPAH in the BWWAF ranged from 0.07 mg/L to 0.17 mg/L, with a response threshold of 0.15 mg/L. For the CEWAF, LC50 values for TPAH were higher, ranging from 0.21 mg/L to 0.73 mg/L, with a response threshold of 0.31 mg/L for the combined dataset (Table 3). There was no statistically significant difference between the CEWAF and BWWAF preparations based LC50 values for parent naphthalene. The mean LC50 was 0.026 mg/L (±0.016 SD) for the CEWAF and 0.050 mg/L (±0.034 SD) for the BWWAF (Table 4).

**Table 4:** Summary of acute toxicity results for three arctic species based on parent naphthalene concentrations measured at test initiation

Test	*Calanus glacialis*			*Calanus glacialis*			*Boreogadus saida*			*Myoxocephalus* sp		
	Early season			Late season								
	12-d LC50 (mg/L)			12-d LC50 (mg/L)			96-h LC50 (mg/L)			96-h LC50 (mg/L)		
	WAF	BWWAF	CEWAF	WAF	BWWAF	CEWAF	WAF	BWWAF	CEWAF	WAF	BWWAF	CEWAF
1	NC	0.04	0.020	NC	NT	0.014	0.032	0.03	0.171	0.026	0.026	0.024
2	NC	0.031	0.024	NC	NT	0.067	0.035	0.035	0.046	0.035	0.042	0.025
3	NC	0.032	0.048	NC	NT	0.084	0.022	0.029	0.058	0.039	0.052	0.062
4	NC	NC	0.041	NC	NT	0.030	NT	NT	0.071	0.039	0.056	0.069
5	NC	0.111	0.008	NC	NT	0.077						
6	NC	0.035	0.015									
Mean	NC	0.050	0.026	NC	NT	0.054	0.03	0.031	0.087	0.035	0.044	0.035
SD		0.034	0.016			0.031	0.007	0.003	0.057	0.006	0.013	0.024

WAF = water-accommodated fraction; BWWAF = breaking wave water-accommodated fraction; CEWAF = chemically enhanced water-accommodated fraction; LC50 = median lethal concentration; SD = standard deviation; NC = not calculable, undiluted WAF did not show significant toxicity; NT = not tested.

A dispersant-only treatment was evaluated with the early season copepod test series. Toxicity was observed in each dispersant-only treatment, with LC50 values of 17 mg/L and 50 mg/L based on nominal Corexit 9500 concentrations (Table 5). For comparison with dispersed oil treatments, the amount of dispersant predicted to be in the LC50 of CEWAF was 1.1 mg/L to 3.1 mg/L, assuming a dispersant-to-oil ratio of 1:20. This is more than 5-fold lower than the concentration of dispersant predicted to cause mortality.

**Table 5:** Mean survival and median lethal concentration (LC50) data for early-season copepods (*Calanus glacialis*) exposed to Corexit 9500 during a 12-d test

Nominal (%)	Corexit 9500 (mg/L)	4 d		12 d	
		Mean % survival	SD	Mean % survival	SD
Test 1					
Control	0	100	0	95	6
6.25	31	95	10	80	14
12.5	63	92.5	10	30[Table-fn tf5-1]	24
25	100	40	41	0[Table-fn tf5-1]	0
50	250	0	0	0[Table-fn tf5-1]	0
100	500	0	0	0[Table-fn tf5-1]	0
LC50 (mg/L)				50	
Test 2					
Control	0	100	0	95	6
6.25	31	62.5	15	12.5[Table-fn tf5-1]	5
12.5	63	40	18	2.5[Table-fn tf5-1]	5
25	100	5	10	0[Table-fn tf5-1]	0
50	250	0	0	0[Table-fn tf5-1]	0
100	500	0	0	0[Table-fn tf5-1]	0
LC50 (mg/L)				17	

Significantly different than control.

SD = standard deviation.

### Calanus glacialis: Late ice-free season

**Figure 5:**
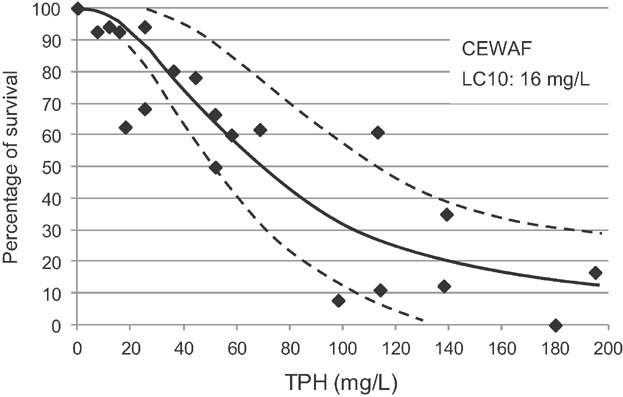
Concentration response curves for late season copepod tests with chemically dispersed Alaska North Slope crude oil (chemically enhanced water-accommodated fractions [CEWAF]; *r*^2^ = 0.89) (control normalized data; dashed lines represent 95% confidence intervals).

No statistically significant differences in survival were observed between the 100% WAF treatments and controls. The concentrations of TPH in the WAF preparations were below 1.0 mg/L. Statistically significant mortality was observed in each of the 100% CEWAF preparations, with corresponding increases in hydrocarbon exposures in these test media. Based on WAF dilution, the mean lethal dilution was 25.9% CEWAF (±10 SD). Based on measured TPH, the mean LC50 was 62 mg/L TPH (±21 SD), with an LC10 response threshold of 16 mg/L TPH based on all data from 5 tests with CEWAF (Figure 5).

The LC50 for TPAH in CEWAF ranged from 0.37 to 1.81 mg/L, with a response threshold concentration of 0.15 mg/L TPAH for the combined dataset. The mean LC50 for the CEWAF was 0.054 mg/L (±0.031 SD) based on parent naphthalene concentrations (Table 5).

### Arctic cod: *Boreogadus saida*

**Figure 6:**
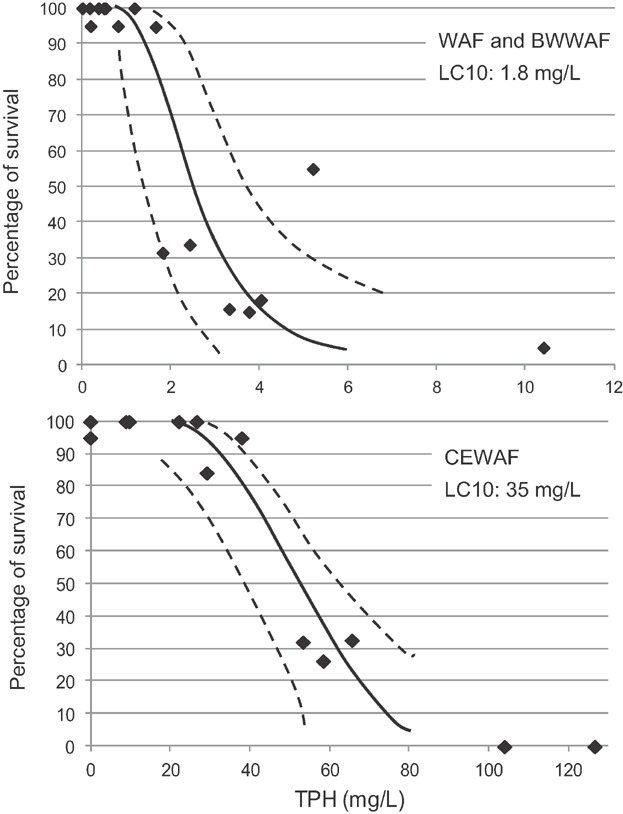
Concentration response curves for Arctic cod tests with physically dispersed (water-accommodated fraction [WAF] and breaking wave WAF [BWWAF]; *r*^2^ = 0.93) and chemically dispersed Alaska North Slope (ANS) crude oil (chemically enhanced WAF [CEWAF]; *r*^2^ = 0.94) (control normalized data; dashed lines represent 95% confidence intervals).

A dose–response was observed for juvenile Arctic cod exposed to both physically-dispersed preparations, with statistically significant decreases in mean percentage of survival for each of the 100% WAF and BWWAF treatments. The mean lethal dilution for the WAF and BWWAF treatments were 51.8% (±7.2 SD) and 37.6.0% (±24.0 SD), respectively (Table 2). Based on TPH, the mean LC50 values for the WAF and BWWAF were 1.6 mg/L (±0.4 SD) and 3.3 mg/L TPH (±2.2 SD), respectively. Using a compilation of data generated from 3 tests with WAF and BWWAF, the LC50 was 2.5 mg/L TPH, and the LC10 response threshold was 1.8 mg/L TPH for the physical dispersions (Figure 6).

Statistically significant decreases in survival were observed in each of the 100% CEWAF treatments. The mean lethal dilution was 13.0% CEWAF (±3 SD), whereas the mean LC50 for the 4 tests was 55 mg/L (±17 SD) based on TPH with an LC10 response threshold of 35 mg/L (Figure 6). As with the copepods, the 96-h LC50 values and response thresholds for the physically-dispersed petroleum were lower than those of the chemically-dispersed petroleum per unit TPH. The mean LC50s for WAF and BWWAF exposures were more than 20 times lower than CEWAF LC50s.

The LC50 for TPAH (Table 3; based on the sum of parent and alkylated PAHs) ranged from 0.05 mg/L to 0.15 mg/L for the WAF and BWWAF preparations, with a response threshold of 0.06 mg/L. The LC50 for the CEWAF ranged from 1.07 mg/L to 3.05 mg/L TPAH, with a response threshold of 1.0 mg/L TPAH for the combined data. The mean LC50 based on parent naphthalene concentration was 0.030 mg/L (±0.007 SD) and 0.031 mg/L (±0.003 SD) for WAF and BWWAF, respectively; whereas the mean LC50 for CEWAF was 0.087 μg/L (±0.057 SD; Table 5). In general, CEWAF treatments produced less toxicity (a higher LC50) per unit TPAH.

### Sculpin: Myoxocephalus sp. 

**Figure 7:**
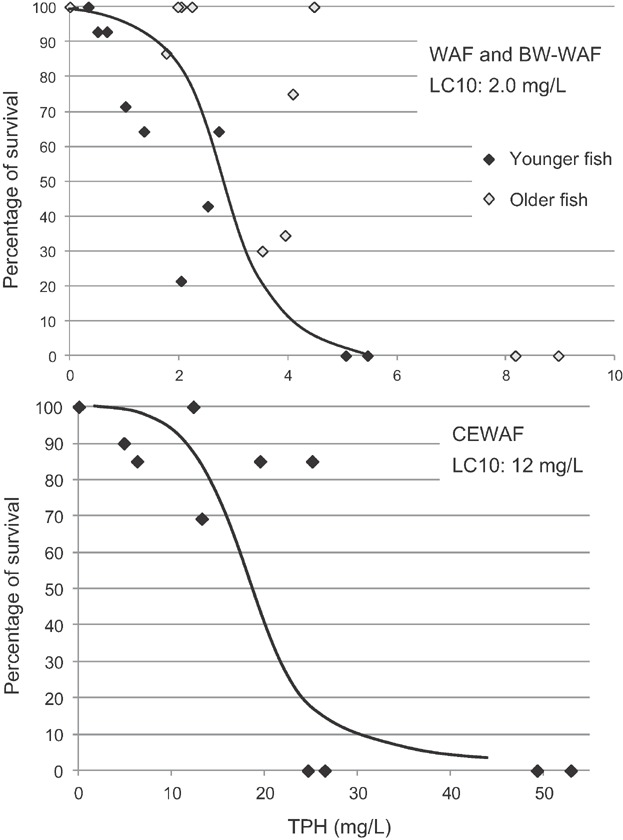
Concentration response curves for sculpin tests with physically dispersed (water-accommodated fraction [WAF] and breaking wave WAF [BWWAF]; *r*^2^ = 0.72) and chemically dispersed Alaska North Slope (ANS) crude oil (chemically enhanced WAF [CEWAF]; *r*^2^ = 0.83) (control normalized data).

Toxicity to larval sculpin was observed in both physically and chemically dispersed oil treatments. Mean percentage of survival in the 100% WAF and BWWAF preparations was significantly less than controls, with a mean lethal dilution of 85.1% (±10.3 SD) and 56.6% (±4.0 SD), respectively. The mean LC50 was 2.3 mg/L (±1.0 SD) and 4.0 mg/L TPH (±1.7 SD) for the WAF and BWWAF, respectively. Based on the combined data set for the physically dispersed WAF and BWWAF, the LC10 response threshold was 2.5 mg/L TPH (Figure 7). Significant increases in exposure concentration and toxicity were observed in the CEWAF treatments, with a CEWAF mean lethal dilution of 5.8% (±2.0 SD) based on WAF dilutions. Based on measured TPH, the mean LC50 was 28 mg/L TPH (±14 SD), with the LC10 response threshold of 12 mg/L (Figure 7). Younger larval fish (∼14 d posthatch) appeared to be more sensitive than older larvae (∼28 d posthatch) when comparing measured concentrations of TPH. For the CEWAF preparations, the mean LC50 for the 14 d posthatch and 28 d posthatch larvae were 17.5 mg/L and 37.5 mg/L TPH, respectively.

The LC50 for TPAH ranged from 0.04 mg/L to 0.08 mg/L and 0.07 mg/L to 0.18 mg/L for WAF and BWWAF treatments, respectively and from 0.32 mg/L to 1.6 mg/L for the CEWAF. In addition, no significant differences were observed between the CEWAF and physical dispersions based on parent naphthalene concentrations. The mean LC50 values for WAF and BWWAF treatments were 0.035 mg/L (±0.006 SD) and 0.044 mg/L (±0.013 SD), respectively; for CEWAF treatments the mean LC50 was 0.035 mg/L (±0.024 SD). There appeared to be a change in sensitivity between larval age groups: the 14 d posthatch (in Tests 1 and 2) were more sensitive than the 28 d posthatch larval sculpin (in Tests 3 and 4; Tables2 and 5).

## DISCUSSION

Three WAFs of oil and a dispersant-only treatment were used to evaluate the relative toxicity of physically and chemically dispersed crude oil to 3 wild-caught arctic species under realistic Arctic conditions using spiked and declining exposures and test durations suitable to the species being tested. The WAF exposure media was intended to represent an untreated, physical dispersion of crude oil similar to those established by Anderson et al. 58, Blenkinsopp et al. 59, and the CROSERF program 4. The BWWAF preparation included a unique mixing step, which periodically introduced increased mixing energy and represented physical dispersions generated by wind and wave conditions at a Beaufort scale of 2 to 3. The CEWAF used similar preparation methods as previous investigators 31,34. Dispersant-only toxicity tests were also performed on wild-caught copepods; these experiments enabled assessment of the potential contribution of dispersant compounds to toxicity of the CEWAF treatments and the toxicity of the dispersant in the absence of oil.

### Chemical composition of the physical and chemical dispersions

Under low Arctic temperatures, fresh ANS crude oil was pourable in the laboratory, but did not move easily into solution with low-energy mixing at a loading rate of 10 g/L fresh ANS. Concentrations of petroleum in the WAF preparations ranged from <1 mg/L to 4 mg/L TPH. Introducing increased mixing energy during the BWWAF preparation increased the amount of hydrocarbons in test solutions, as well as some small oil globules suspended in the water column. Total petroleum hydrocarbon concentrations in the BWWAF increased 3-fold and ranged between 4 mg/L and 13 mg/L TPH. Adding the dispersant dramatically increased the amount of hydrocarbons producing TPH concentrations ranging from 138 mg/L to 1180 mg/L. This was significantly more than the physical dispersions. These results are similar to a previous study of physically and chemically dispersed oil in cold temperatures where TPH concentrations in CEWAF prepared with fresh ANS crude oil were also significantly higher than those of WAF preparations at 7 °C 60.

**Figure 8:**
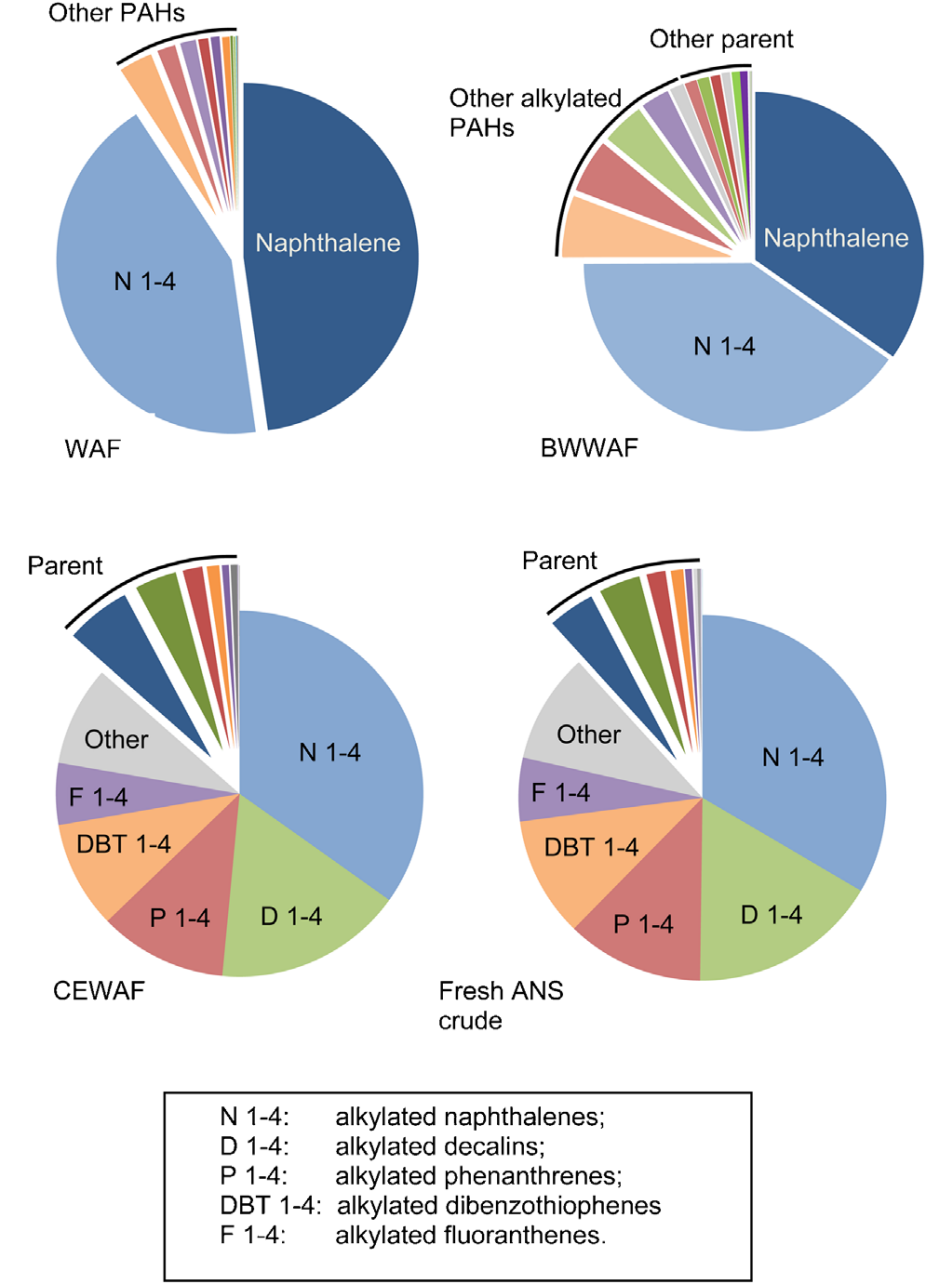
Proportion of parent and alkylated polycyclic aromatic hydrocarbons (PAH) in undiluted water-accommodated fractions (WAF) of Alaska North Slope (ANS) crude oil. BWWAF = breaking wave WAF; CEWAF = chemically enhanced WAF.

The composition of PAHs for the physical dispersions (WAF and BWWAF) was consistent among test stock solutions and was dominated by naphthalene and alkylated naphthalenes (75–90% of the TPAH; Figure 8). Adding dispersant dramatically shifted the composition of the PAHs. With the exception of naphthalene and bicyclic decalin (7% and 5% of the TPAH, respectively), parent compounds accounted for a very small percentage of the PAHs in the CEWAF (Figure 8). The alkylated naphthalenes accounted for one-third of the TPAH, with alkylated decalins, fluorenes, phenanthrenes, and dibenzothiophenes representing an additional 65%. The PAH distribution in the CEWAF was very similar to that of fresh ANS crude, which was also dominated by the alkyl homologs of low molecular weight PAHs. Both the CEWAF and crude ANS had low concentrations of high molecular weight PAH compounds. Couillard et al. 61 also observed that the relative proportion of PAHs was altered with the addition of dispersants, with a shift toward alkyl homologs. They also found that the PAH composition in the dispersed WAF was very similar to that of the crude petroleum used in their study. The work of Couillard et al. 61 and the findings of the present study suggest that the similarity in PAH composition between the CEWAF and the crude oil was presumably because of the formation of neutrally buoyant small droplets released into the water column by the dispersant. Although there was also an increase in the more soluble hydrocarbons (e.g., parent naphthalene) with the addition of dispersant, the less soluble hydrocarbons associated with oil droplets account for the majority of the TPH and PAHs present in the CEWAF (Table 1).

### Relative sensitivity of Arctic species

The open-water season in the Beaufort and Chukchi Seas generally extends from July through November. During this time, the composition of pelagic species changes dramatically with increased phytoplankton and zooplankton abundance as ice breaks up and is followed by an influx of fish and mammalian species taking advantage of the increased food resources. During this relatively short season, species such as copepods and fish undergo rapid growth phases and changes in physiology 19,20. To address potential changes in sensitivity during the open-water season, copepods were tested in both the early and late seasons. Toxicity testing with fish included different age classes, late-yolk-sac stage and young-of-the-year juveniles. The 3 Arctic species tested under this experimental program showed remarkable similarity in sensitivity within each of the various types of oil elutriates.

For the physically dispersed petroleum (WAF and BWWAF combined), the mean LC50 values representing all species ranged from 1.6 TPH/L to 4.0 mg TPH/L (Table 2). For the chemically dispersed petroleum, the early season copepods and the larval sculpin had mean LC50s of 22 (±10 SD) mg/L and 28 (±14 SD) mg/L TPH. The sensitivity of the late-season copepods was similar to the Arctic cod, with mean LC50s of 62 mg/L (±21 SD) and 55 mg/L TPH (±17 SD), respectively, for CEWAF treatments. Overall, the range of LC50s for CEWAF was 14 mg/L to 80 mg/L TPH, which is a relatively small range for different phyla and age classes. Tests conducted under the CROSERF program with 9 species representing 3 phyla from larval to adult age classes had a similarly narrow range of responses (3–49 mg/L TPH) 4. These findings are consistent with another recent study that found that a variety of Arctic and non-Arctic species exhibited a similar sensitivity to WAF, parent naphthalene, and methyl-naphthalene 62.

Based on measured TPH concentrations, the copepod LC50s for the CEWAF were significantly lower in the early season compared to the late-season tests. The mean CEWAF LC50s for early-season copepods tested in the early season was 22 mg TPH/L, with a range from 14 TPH/L to 37 mg TPH/L. The mean CEWAF LC50 for the late-season copepods was 62 mg/L, with a range from 30 mg/L to 79 mg/L TPH. This decrease in apparent sensitivity is presumed to be related to differences in size or developmental stage 63; however, physiological changes such as increasing lipid stores prior to winter may increase the copepods' tolerance in the late season 19,20. The mean LC50 (28 mg/L TPH) for larval sculpin were significantly lower than for the juvenile Arctic cod (55 mg/L TPH). However, the mean LC50 for the late-yolk-sac stage was 17.5 mg/L TPH (±1.0 SD), whereas the mean LC50 for the older cohort was 37.5 mg/L TPH (±12 SD) and was similar to juvenile cod. This may be indicative of the changes in sensitivity that occur during the rapid larval development that Arctic species undergo during the short open-water season.

### Relative toxicity of physically and chemically dispersed petroleum

Although the use of chemical dispersant significantly increased petroleum concentrations in test solutions, the acute toxicity of the physically-dispersed petroleum was greater than that of the chemically dispersed petroleum when expressed in terms of measured TPH or TPAH concentrations (i.e., LC50s were lower for the WAF and BWWAF than for the CEWAF). In copepod tests, the LC50s for the BWWAF preparation were 3- to 9-fold lower than those of the CEWAF when expressed as TPH concentration. Similarly, LC50s for the physically dispersed preparations were 9 times to 32 times lower than those for the CEWAF for the experiments run with fish when based on TPH concentrations. Chemical dispersants reduce the surface tension between oil and water molecules, facilitating the movement of oil into the aqueous phase; most of the oil is associated with droplets and is not in a freely dissolved phase 43. As a result, these components are expected to be in a less bioavailable, acutely toxic form. Fuller et al. 5 reported a similar relationship, suggesting that the bulk of the measured hydrocarbons in CEWAF are in the form of colloidal micelles that move through fish gills without being taken up by test organisms, whereas the WAF is dominated by dissolved hydrocarbons that are more readily absorbed into tissues.

These findings are consistent with studies conducted with non-Arctic species. The CROSERF program generated LC50s for spiked exposures to WAF and CEWAF preparations for 7 species over a range of sub-Arctic and temperate conditions. The CROSERF experiments were conducted by a variety of laboratories using a spiked exposure with fresh crude oil; however, the experiments were conducted in a sealed system, which maximized the exposure to the more volatile fractions 4. With the exception of topsmelt (*Atherinops affinis*), the mean LC50 values for physically dispersed petroleum based on the TPH concentrations were lower than those of the chemically dispersed petroleum. Water-accommodated fractions were 1.3 times to 36 times more toxic than CEWAF, with the greatest WAF toxicity occurring in exposures with larval turbot (*Scophthalmus maximus*). Two common laboratory test species, the mysid (*Americamysis bahia*) and the Inland silverside (*Menidia beryllina*) also had lower LC50 ranges from WAF exposures compared with CEWAF exposures.

When toxicity was expressed as percentage of dilution of the WAF solution (nominal concentrations), the CEWAF exposures appeared to be more toxic than WAF and BWWAF. However, results expressed in this manner do not represent actual concentrations of petroleum hydrocarbons. Other researchers have found similar discrepancies between the results expressed as nominal loadings or WAF dilutions rather than measured concentrations 4,31,43,64, indicating that the manner in which test results are expressed is an important consideration for risk evaluations and decisions made by spill-response command posts. Expressing toxicity data as measured concentrations facilitates comparison to field observations, which can, in turn, support risk evaluations such as net environmental benefits analysis. Clark et al. 31 suggested that failing to relate toxicity with measured exposure concentrations can lead to erroneous management decisions, such as selecting a dispersant that appears to be less toxic, but is actually less effective at dispersing oil into the water column or at mitigating surface and shoreline impacts.

### Toxicity of Corexit 9500

The toxicity of Corexit 9500 prepared in a similar way to the other WAFs but in the absence of petroleum was evaluated during the experiment conducted with copepods from the early season field collection; LC50 values ranged from 17 mg/L to 50 mg/L. This is similar to responses observed for several invertebrate and fish species exposed to Corexit 9500 in spiked exposures. Aurand et al. 65 exposed early life stages of the temperate estuarine copepod, *Eurytoma affinis* to Corexit 9500. The younger 24-h-old and 48-h-old copepods were more sensitive than the Arctic copepods, with LC50 values of 6.3 mg/L to 14.6 mg/L. Median lethal concentrations reported for mysids vary considerably. Hemmer et al. 66 and the USEPA 67 reported LC50s ranged from 25 mg/L to 130 mg/L; however, Singer et al. 68 and Fuller and Bonner 69 reported LC50 concentrations at much higher levels ranging from 158 mg/L to 1305 mg/L. The LC50 for fish tested under temperate conditions ranged from 25.2 mg/L to 130 mg/L for *M. beryllina* 5,66,70 and was 670 mg/L for *Cyprinodon variegatus* 69.

Based on the dispersant loading rate of 0.5 g/L used in the CEWAF preparations for the present study, the concentration of Corexit 9500 in the mean lethal concentration of CEWAF was calculated to be approximately 1.1 mg/L. This is well below the range of dispersant-only LC50 values for *C. glacialis* obtained during the present study (17–50 mg/L). The facts that chemically dispersed petroleum produced less toxicity per unit of measured TPH concentration and the calculated concentration of the dispersant in the mixture was at least 20-fold lower than the effects level indicates that the dispersant did not contribute to the acute toxicity of CEWAF. This finding is consistent with studies conducted with temperate mysids and fish 3,5 and the mullet, *Liza ramada* 71. Dispersant concentrations in the field have been estimated to be 0.030 mg/L immediately following application 72. This is 3 orders of magnitude below levels predicted to be acutely toxic to copepods, indicating that the dispersant would not be predicted to be acutely toxic for the environmental applications of Corexit 9500. The National Research Council also noted, “there is seldom evidence for synergism (i.e., greater than additive toxicity) between oil and dispersant components, validating the general conclusion that oil is as acutely toxic as dispersed oil” 43.

### The role of parent and alkylated PAHs

**Figure 9:**
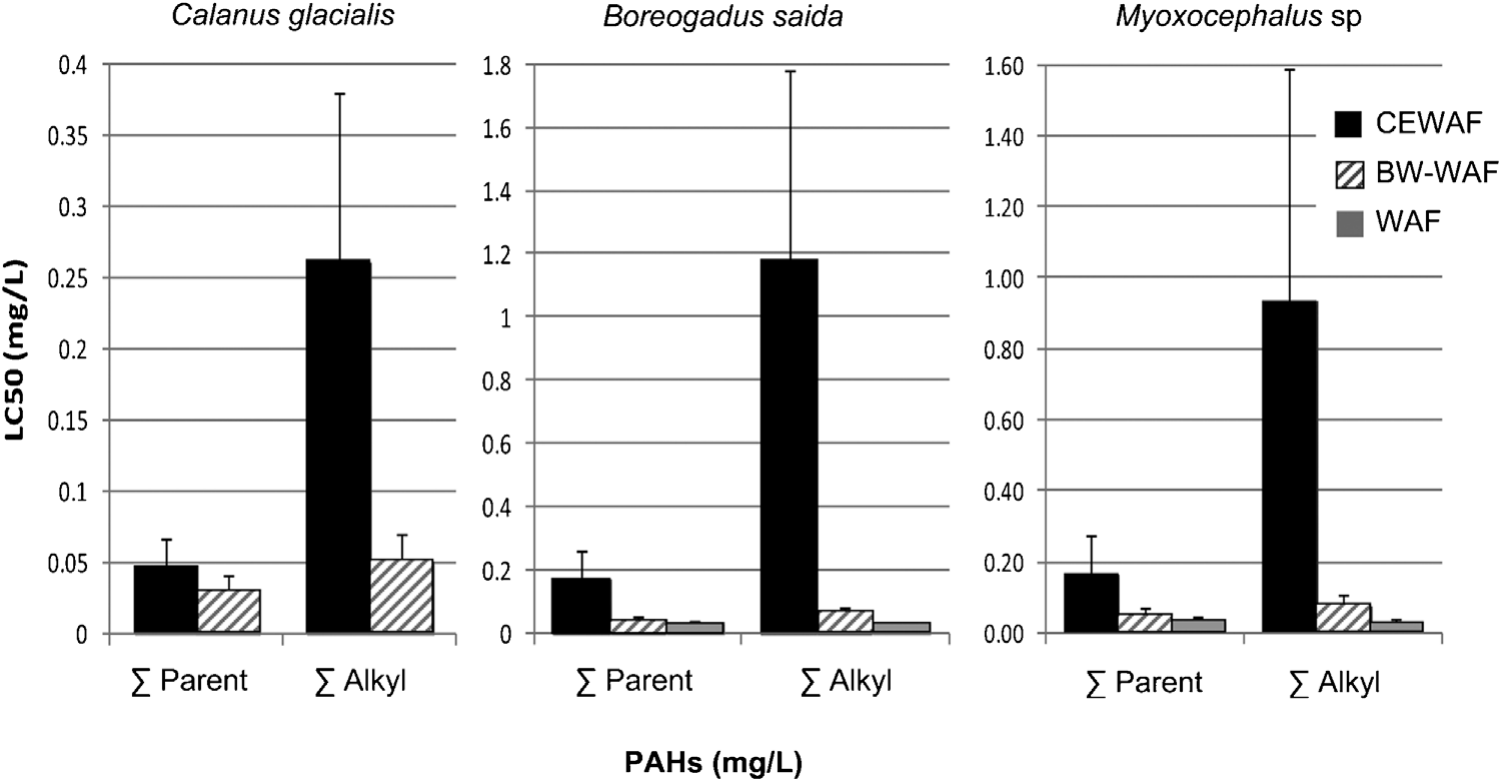
Comparison of parent and alkylated polycyclic aromatic hydrocarbons (PAH) median lethal concentrations results for chemically enhanced water-accommodated fraction (CEWAF), breaking wave WAF (BWWAF), and WAF exposure to 3 Arctic species (error bars represent 95% confidence interval).

The least variable predictor of toxicity for all accommodated fraction preparations for all of the test species were the parent PAH compounds; more specifically, parent naphthalene. The LC50 values for the parent PAHs were similar despite substantial differences in concentrations between the chemical and physical dispersions. Conversely, the acute toxicity of alkylated PAHs was consistently lower in the CEWAF despite having concentrations that were substantially higher than in the WAF and BWWAF (Figure 9).

**Figure 10:**
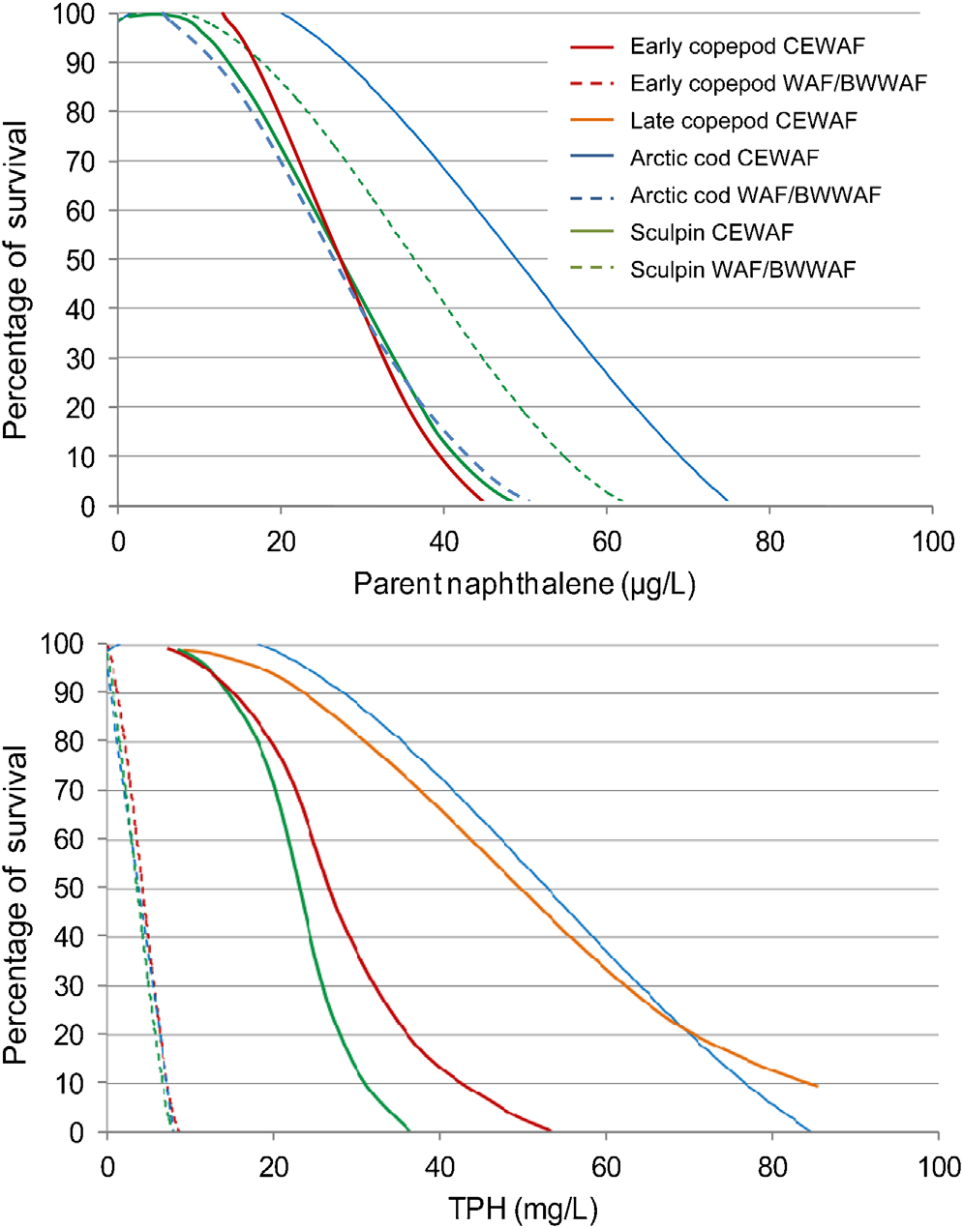
Comparison of concentration response curves for (A) parent naphthalene, and (B) total petroleum hydrocarbons. WAF = water-accommodated fractions; BWWAF = breaking wave WAF; CEWAF = chemically enhanced WAF.

For parent naphthalene, no significant difference was observed between the mean LC50 concentrations for physical and chemical dispersions, even though concentrations produced in the accommodated fractions were 8 times to 11 times higher in the CEWAF dispersions (Table 1; Figure 2). The LC50 values for the WAF, BWWAF, and CEWAF exposures to each of the Arctic species and life stages evaluated with the present experiments were within a factor of 2, ranging from 0.026 mg/L to 0.054 mg/L naphthalene (Table 5 and Figure 10A). In contrast, the mean TPH LC50s were 1.6 mg/L to 55 mg/L TPH for physical and chemical dispersions respectively, a 34-fold difference (Table 2 and Figure 10B). A similar relationship was found for physically and chemically dispersed petroleum based on data generated by CROSERF 4 and Neff et al. 73. During the CROSERF study, parent naphthalene concentrations were measured in CEWAF made with Prudhoe Bay crude and Venezuelan crude, which were then tested in spiked exposures. The LC50 values for the fish species *Sciaenops ocellatus* (Red drum), *Menidia beryllina* (Inland silverside), and *Atherinops affinis* (topsmelt) ranged from 0.067 mg/L to 0.131 mg/L parent naphthalene. For the mysids, *A. bahia* and *Holmesmysis costata*, the parent naphthalene LC50s ranged from 0.050 mg/L to 0.219 mg/L. In spiked exposures to WAF for both temperate and tropical species 73,74, the LC50s for the fish *Amphiprion clarkii* (Clark's clownfish) and *M. beryllina* were 0.039 mg/L to 0.093 mg/L parent naphthalene. For the shrimp *Penaus vannamei* and the mysid *A. bahia* the LC50s were 0.032 mg/L to 0.132 mg/L parent naphthalene. As in the present study, the CROSERF program found that acute toxicity expressed as TPH varied considerably, with mean LC50 in CEWAF preparations higher than those for the WAF.

Naphthalene and alkylated naphthalenes have been associated with the acute toxicity of physically and chemically dispersed petroleum 47,75–77. This has typically been attributed to narcosis and disruption of cellular function associated with uptake of the more soluble, dissolved PAH components 78. In spiked exposures to physically and chemically dispersed petroleum, the less soluble alkyl naphthalene homologs predominate but do not attain a steady state in the test organisms over the short time of the acute exposures 79,80. The declining exposure concentration and the reduced time for development of steady state tissue concentrations suggest that this form of narcosis toxicity may not fully develop during acute experiments.

The narrow range of LC50s in the test waters for parent naphthalene in chemically and physically dispersed petroleum indicates that the dispersant and the presence of oil droplets did not change the acute toxicity of parent naphthalene. This suggests that the dispersant is not altering the toxicity of the oil or the underlying mechanism of toxicity in the spiked exposures, but rather enhancing the absolute concentration of more soluble, dissolved hydrocarbons in the test solutions that are correlated most closely to acute toxicity. Furthermore, particulate oil or alkylated PAHs in the test waters did not appear to add measurably to the toxicity beyond that associated with parent naphthalene concentrations in the physical and chemical dispersions of these oils.

### Comparison with petroleum hydrocarbon concentrations measured in field trials

Mechanical recovery is often considered one of the preferred spill response options in the Arctic. Certain conditions, however, such as the magnitude of release, remote locations, encounter rate of collecting methods, spilled oil, or inclement weather can limit its effectiveness. Typically, dispersants are applied to treat large spill areas in order to increase biodegradation potential and limit the risks to sensitive species living near the waters' surface including birds, whales, and seals and to prevent extensive oil stranding on shorelines. Optimizing use of dispersants is based on the premise that after initial dilution into the water column, turbulent diffusion and mixing will minimize the exposure concentrations to water column species.

Field trials conducted with a variety of oil types and Corexit 9500 show that within the first several hours, dispersed oil concentrations diminish with depth and time to concentrations below the LC10 threshold concentrations observed in the present study (12–35 mg/L TPH). In field trials in the North Sea, approximately 95 barrels of Troll crude (American Petroleum Institute [API]: 30–40°) were dispersed with Corexit 9500 with wave heights of 1 m to 2 m and wind speeds of 1.8 m/s to 11 m/s 81. Surface (∼1 m depth) concentrations were approximately 23 mg/L TPH immediately after treatment, dropping to 15 mg/L within 15 min (Figure 1). At 5 m depth, TPH concentrations were 5.8 mg/L immediately after application and decreased to 1.9 mg/L within 15 min. At 10 m depth, the initial TPH concentration was 1.5 mg/L, declining to 0.062 mg/L within 45 min. Field trials with Prudhoe Bay crude oil (API: 28°) and Corexit showed a similar trend, with initial TPH concentrations at the surface decreasing from 32 mg/L to 13 mg/L within 1 h and to 0.85 mg/L TPH after 3.6 h under a Beaufort sea state of 2 to 3 82,83. At a depth of 5 m, TPH concentrations were 12 mg/L, 15 mg/L, and 2.5 mg/L over the same exposure periods. Concentrations at 9 m were <0.07 mg/L, 1.5 mg/L, and 0.5 mg/L TPH. Rapid dilution was also observed with trials with Murban (API: 39°) and La Rosa crude (API: 24°). Field toxicity tests with copepods and bacteria (Microtox) conducted during field trials on the North Sea showed similar results. Coelho et al. 84 found statistically significant effects at depths up to 5 m, but only when concentrations were still elevated during initial sampling. Within 15 min to 1 h, concentrations were reduced to below acute toxicity effect levels. Dispersants were used extensively to limit shoreline impacts following the Sea Empress oil spill, which released approximately 72 000 tons of North Sea crude oil. Batten et al. 85 reviewed long-term plankton records for the southern Irish Sea before and after the event to ascertain the toxic impact to the plankton. The authors concluded that most common taxa showed no significant changes.

**Figure 11:**
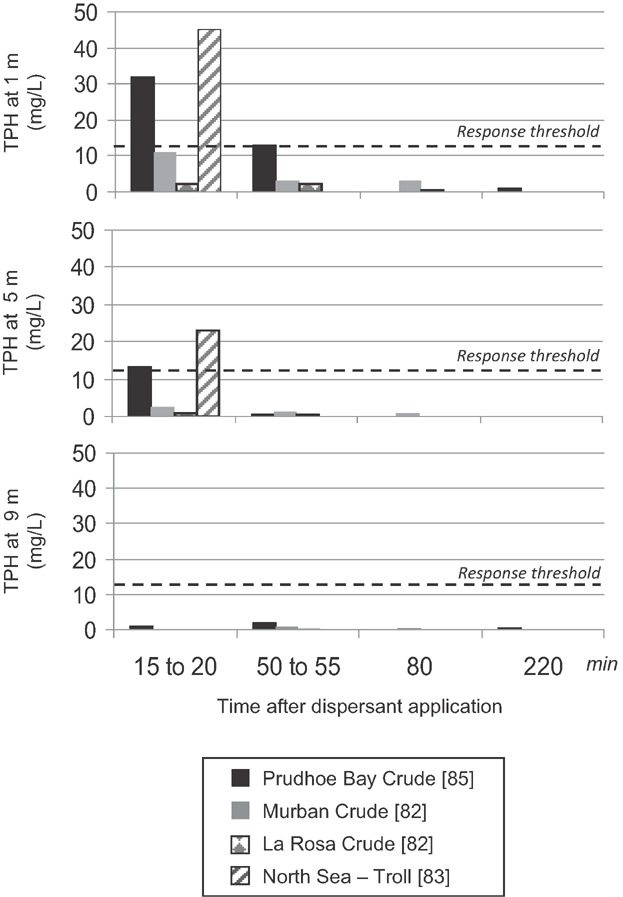
Predicted dilution of total petroleum hydrocarbons (TPH) concentrations by water depth and time. Response threshold values defined as the lowest LC10 for tested arctic species noted by dotted line.

Based on these field trials, TPH concentrations may exceed acute toxicity effect thresholds based on ANS dispersed crude oil toxicity test results immediately following dispersant application in open water (Figure 1). However, based on earlier field studies, water column dilution limits toxic concentrations to only the first few meters of the water column. Subsequent turbulent mixing will likely reduce concentrations to below acute toxicity threshold concentrations for the Arctic species examined in the present study in hours to minutes depending on distance from the surface. In separate publications, data generated from the present study will also be used to compare Arctic species to non-Arctic species and as data input for quantifying oil spill impacts using population models.

## CONCLUSIONS

Acute toxicity tests were conducted with 3 pelagic arctic species under open-water, ice-free conditions of the Beaufort and Chukchi Seas. Although TPH concentrations were predictably higher in the CEWAF preparations, the physically-dispersed ANS crude oil was more acutely toxic when calculated as per unit TPH. However, when expressed as parent PAHs, specifically parent naphthalene, lethality demonstrated by the 3 Arctic species was similar among the physically and chemically dispersed preparations. The commonality of the acute response to naphthalene has been observed in data from previous studies using spiked exposures with a variety of species and oil types 4,70. The fact that the LC50s were similar when expressed in terms of parent naphthalene indicated that parent naphthalene was a better proxy of dissolved toxic units than TPH or percentage of WAF for assessing the acute toxicity of petroleum. The similarity in parent naphthalene toxicity for the physically and chemically dispersed ANS indicated that Corexit 9500 was not contributing to the adverse effects observed. Instead, it was enhancing the absolute concentration of hydrocarbons removed from the surface and distributed within the water column. Furthermore, the underlying mechanism of acute toxicity of dispersed petroleum hydrocarbons are not altered by the chemical dispersant.

The effects thresholds as defined by the LC10 in the present study were above TPH concentrations predicted to occur in the field within hours of surface application of dispersants. Thoroughly considering the environmental risks to Arctic resources needs to consider the relative value of moving oil from the surface of the water into the pelagic environment. The various environmental resources exposed within the environmental compartments at the sea surface, the water column, and the neighboring shorelines need to be evaluated when comparing response options to an oil spill. The data provided in the present study can be used to support an ecosystem consequence analysis.

## SUPPLEMENTAL DATA

**Table S1.** (13 KB XLS).

## Supporting Information

All Supplemental Data may be found in the online version of this article.
